# A Pipeline for Volume Electron Microscopy of the *Caenorhabditis elegans* Nervous System

**DOI:** 10.3389/fncir.2018.00094

**Published:** 2018-11-21

**Authors:** Ben Mulcahy, Daniel Witvliet, Douglas Holmyard, James Mitchell, Andrew D. Chisholm, Yaron Meirovitch, Aravinthan D. T. Samuel, Mei Zhen

**Affiliations:** ^1^Lunenfeld-Tanenbaum Research Institute, Mount Sinai Hospital, Toronto, ON, Canada; ^2^Department of Molecular Genetics, University of Toronto, Toronto, ON, Canada; ^3^Department of Pathology and Laboratory Medicine, Mount Sinai Hospital, Toronto, ON, Canada; ^4^Nanoscale Biomedical Imaging Facility, The Hospital for Sick Children, Peter Gilgan Centre for Research and Learning, Toronto, ON, Canada; ^5^Center for Brain Science, Department of Physics, Harvard University, Cambridge, MA, United States; ^6^Section of Cell and Developmental Biology, Division of Biological Sciences, University of California, San Diego, La Jolla, CA, United States; ^7^Computer Science and Artificial Intelligence Laboratory, MIT, Cambridge, MA, United States; ^8^Department of Physiology, University of Toronto, Toronto, ON, Canada; ^9^Department of Cell and Systems Biology, University of Toronto, Toronto, ON, Canada

**Keywords:** *C. elegans*, volume electron microscopy, connectome, nervous system, high-pressure freezing

## Abstract

The “connectome,” a comprehensive wiring diagram of synaptic connectivity, is achieved through volume electron microscopy (vEM) analysis of an entire nervous system and all associated non-neuronal tissues. White et al. (1986) pioneered the fully manual reconstruction of a connectome using *Caenorhabditis elegans*. Recent advances in vEM allow mapping new *C. elegans* connectomes with increased throughput, and reduced subjectivity. Current vEM studies aim to not only fill the remaining gaps in the original connectome, but also address fundamental questions including how the connectome changes during development, the nature of individuality, sexual dimorphism, and how genetic and environmental factors regulate connectivity. Here we describe our current vEM pipeline and projected improvements for the study of the *C. elegans* nervous system and beyond.

## A Brief Background of *Caenorhabditis elegans* Connectomics

In the 1960s, Sydney Brenner and colleagues adopted the nematode *Caenorhabditis elegans* as a model to better understand the development and function of a complete nervous system. Part of their strategy was to reconstruct the entire synaptic wiring diagram of a nervous system using manual volume electron microscopy (vEM). *C. elegans* was a wise choice. Its small size, a cylinder of ∼1 mm in length and 70 μm in diameter, provided a reasonable chance of success with the laborious and technically challenging procedures required for vEM. Nichol Thompson developed the essential skill in cutting long series of serial sections without gaps. Initial successes included reconstructions of the anterior sensory anatomy (Ward et al., 1975; Ware et al., 1975), the pharyngeal nervous system (Albertson and Thomson, 1976), and the ventral nerve cord (White et al., 1976). When John White and Eileen Southgate succeeded in tracing the nerve ring, the first near-complete wiring diagram of an animal’s nervous system was obtained (White et al., 1986; White, 2013). The *C. elegans* connectome provided the first comprehensive physical map through which information flows to select, enact, and modify motor functions. This structural foundation first allowed the formulation and experimental validation of hypotheses for mechanosensory and motor behaviors (Chalfie et al., 1985). The small number of neurons and their connections has since inspired numerous theoretical and experimental studies to model entire sensorimotor circuits (e.g., Varshney et al., 2011; Towlson et al., 2013; Szigeti et al., 2014; others).

With the recent emergence of partial wiring diagrams for neural circuits in other invertebrates and some vertebrates (e.g., Helmstaedter et al., 2013; Takemura et al., 2013; Randel et al., 2014, 2015; Kasthuri et al., 2015; Ryan et al., 2016, 2017; Eichler et al., 2017; Veraszto et al., 2017; Williams et al., 2017; others), the search for conserved features and circuit motifs that might have homologous functions across species becomes possible.

*Caenorhabditis elegans* connectomics will play a crucial role in uncovering general principles of neural circuit structure and function. The *C. elegans* nervous system embeds computational properties sufficiently powerful for many complex behaviors: different motor patterns and states, adaptive, and integrative sensory perception, as well as forms of associative learning and memories (Zhang et al., 2005; Ardiel and Rankin, 2010; Sasakura and Mori, 2013; Allen et al., 2015; Zhen and Samuel, 2015). Its small and accessible size – both in terms of neuron number (302) and synapse number (∼7000) – makes it a tractable system to propose and test theoretical models of nervous system function. If the circuit designs that enable sensory coding, decision-making, and plasticity are evolutionarily conserved, understanding mechanisms of the compact *C. elegans* nervous system will yield useful insights into shared principles.

Progress still needs to be made at multiple fronts in *C. elegans* connectomics.

First, the original *C. elegans* connectome was assembled from partially overlapping fragments of a few individuals, not one intact individual (White et al., 1986). The validity of this approach hinges on the stereotypy of the wiring diagram across individuals. The stereotypy observed for most *C. elegans* cells identified by lineage studies (Sulston and Horvitz, 1977; Sulston et al., 1983) and preliminary comparison of the central nervous system connectivity of two animals (Durbin, 1987) made this plausible. However, an explicit analysis of variability across connectomes of multiple individuals is required.

Second, postembryonic neurogenesis occurs across *C. elegans* development. Post-embryonically born neurons make up ∼25% of neurons in the adult. The original *C. elegans* connectome was assembled from parts of several adults and one last stage larva, reflecting one snapshot of a dynamic wiring diagram. How the connectome develops, remodels to incorporate newly born neurons, and modifies the behavioral repertoire at different developmental stages needs to be addressed.

Third, sexual dimorphism is prominent in the *C. elegans* nervous system. Compared to adult hermaphrodites, adult males have an additional 85 neurons, accounting for ∼20% of the nervous system (Sulston and Horvitz, 1977; Sulston et al., 1980; Sammut et al., 2015; Molina-Garcia et al., 2018). Though progress has been made on the wiring of parts of the male nervous system (Hall and Russell, 1991; Jarrell et al., 2012), a complete and comprehensive side-by-side comparison of high-quality male and hermaphrodite connectomes awaits.

Fourth, natural variants of *C. elegans* exhibit substantial genetic and behavioral differences from that of the laboratory wild-type strains. The connectomes of these and other nematode species should be obtained and compared.

Addressing questions about individual variability, developmental plasticity, sexual dimorphism, genetic perturbations, and so on requires higher-throughput vEM reconstruction. Recent focus on technology development, such as automation in serial sectioning (Schalek et al., 2012), image acquisition (Inkson et al., 2001; Denk and Horstmann, 2004; Holzer et al., 2004; Heymann et al., 2006; Knott et al., 2008; Hayworth et al., 2014), and segmentation of neurons and connections (Saalfeld et al., 2009; Helmstaedter et al., 2011; Cardona et al., 2012; Boergens et al., 2017), has accelerated vEM throughput. Originally designed to allow acquisition of connectomes of single large samples, these technological advances offer small model systems such as *C. elegans* an opportunity to employ vEM as a rapidly deployable tool for developmental and comparative connectomics, and other aspects of nematode biology.

Below we describe such a pipeline.

## Outline of a Pipeline for Current *C. elegans* EM Studies

**FIGURE 1:**
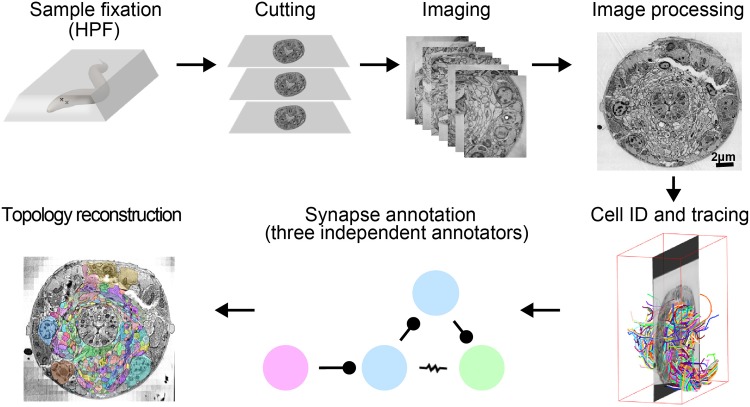
A pipeline for *C. elegans* connectome reconstruction using vEM. Samples are fixed using high-pressure freezing and freeze substitution, embedded in plastic then cut into ultrathin serial sections before imaging on an electron microscope. Images are stitched together into a 3D volume, and neurons are identified and traced throughout the dataset by skeleton tracing using CATMAID. Synapses are annotated by three independent annotators to obtain the connectome. Volumetric reconstruction, which yields topographical information of cells and neurons, is facilitated by computational filling followed by manual proofreading using VAST.

This pipeline has been successfully used for high-throughput volume reconstruction of intact *C. elegans* of all developmental stages, and has yielded high-resolution connectomes for multiple animals (Figure 1; Witvliet et al., in preparation). We describe technical issues general to vEM studies and highlight key technical considerations for *C. elegans*.

### Step 1: Preparing Samples for EM

Rapid freezing of living animals facilitates uniform vitrification. Subsequent freeze-substitution and fixation allows preservation of organelles, cells, and tissues in their native states. Due to its small size, intact *C. elegans* is well suited to high-pressure freezing, circumventing the mechanical damage and physiological perturbation caused by dissection. Through standard *en bloc* and post-sectioning staining with heavy metals, sufficient contrast can be imparted to lipids, proteins, and nucleic acids for visualization with an electron microscope.

### Step 2: Serial Sectioning

The thickness and number of serial sections are determined by the sectioning method, as well as the size of the object of interest. Reducing section thickness facilitates reconstruction of fine cellular structures (such as neurites), and distinction between intracellular features (such as vesicles, ER, and microtubules). Because of the small diameter of *C. elegans* neurites, serial sections of 50 nm or thinner are needed for reliable connectome reconstruction.

### Step 3: Image Acquisition and Processing

Image resolution is set by the size of object of interest. For adult and larval connectome reconstructions, a resolution of 1–2 nm per pixel is optimal for reliable synapse annotation. A montage of images that cover the area of interest are computationally stitched and aligned into a 3D volume. Minimization of artifacts during sample preparation (e.g., mechanical compression during sectioning) and imaging (lens distortion and shrinkage during electron beam exposure), and their correction are critical for acquiring a well-aligned image volume.

### Step 4: Segmentation

The aligned image stacks are segmented into objects of interest. For connectomes this means tracing neurons and mapping synapses. Volumetric segmentation consists of coloring in each section of neurite throughout the volume, reconstituting the 3D morphology of the cell. Skeleton segmentation consists of placing a point in the center of the neurite on each section. Tracing skeletons is faster than volumetric segmentation, but less rich in morphological detail.

### Step 5: Synapse Annotation

Synapse identification is based on stereotypic ultrastructural features. A sample with well-preserved neurite morphology and intracellular organelles, such as presynaptic active zones and synaptic vesicles, facilitates high-confidence annotation of chemical synapses. However, synapse annotation is not completely objective. Subjectivity arises in the identification of small synapses, gap junctions, and assigning postsynaptic partners for polyadic synapses. Increased section thickness, section and staining artifacts, and unfortunate synapse orientation relative to the plane of sectioning also increase subjective uncertainty. Parallel annotation of the same dataset by multiple tracers, constructing connectomes from multiple animals, and comparing with existing datasets help to reduce annotation errors.

### Step 6: Neuron Identification

Every somatic *C. elegans* cell can be assigned a unique name. The location and identity of each nucleus was lineage-mapped by following its migration throughout development (Sulston and Horvitz, 1977; Sulston et al., 1980, 1983; White et al., 1986). Additionally, all processes within the neuropils have characteristic features, allowing identification without necessarily tracing the process back to the cell body. Stereotypic features include entry-point into the neuropil, neurite trajectory and morphology, placement within the neuropil, abundance of clear and dense-core vesicles, multi-synapse clusters, and unique morphological features. Each neuron can be identified by characteristic features at multiple points along its process, increasing the confidence of tracing.

## Step-By-Step Description of Methods and Considerations

### Preparation of EM Samples

#### General Considerations for High-Pressure Freezing and Freeze Substitution

**FIGURE 2:**
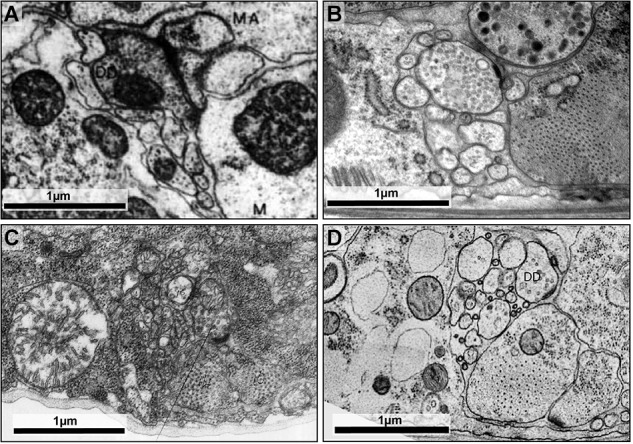
High-pressure freezing improves preservation of ultrastructure. **(A)** The dorsal cord of an adult prepared using the slow chemical fixation protocol (White et al., 1978). The DD motor neuron is making a neuromuscular junction to dorsal muscle cells. **(B)** The dorsal cord of an adult fixed using high-pressure freezing and imaged using TEM. The DD motor neuron is making a neuromuscular junction to dorsal muscle cells. **(C)** The ventral nerve cord of a chemically fixed first stage (L1) *C. elegans* larva (White et al., 1978). The DD axon makes a NMJ to the ventral muscle cell (M). **(D)** A TEM micrograph of the ventral nerve cord of a high-pressure frozen first stage larva (L1) at similar region, where DD makes a NMJ to the ventral muscle cell. The advent of high-pressure freezing allows better preserved neurite morphology, synapse structure, and extracellular space, facilitating connectomic and topological analyses of the *C. elegans* nervous system. Scale bar 1 μm. Panel **(A)** was reprinted with permission from White et al. (1978). Panel **(C)** a scan of the micrograph used in White et al. (1978), hosted by the WormImage Consortium (www.wormimage.org).

For the original *C. elegans* wiring diagram reconstruction, animals were submerged in one or more chemical fixatives, either glutaraldehyde followed by osmium tetroxide, or osmium tetroxide alone (White et al., 1986). Some animals were cut by razors to aid the diffusion of fixatives through the tissue. This fixation process is not instantaneous (e.g., tomato hair cells have been estimated to be fixed at a rate of 2 μm/s in a glutaraldehyde-cacodylate solution; Mersey and McCully, 1978), and distortions to native ultrastructure occur before fixation is complete (Smith and Reese, 1980; Gilkey and Staehelin, 1986; Figures 2A,C).

A better strategy for tissue preservation involves rapid freezing of samples in vitreous ice, dehydration at low temperatures to prevent the growth of damaging ice crystals, and simultaneous fixation. In early work in other experimental systems, this was achieved by subjecting samples to extremely low temperature (around -175°C), either by plunging the sample into cold liquids, propelling the cold liquid at the sample (Feder and Sidman, 1958; Moor et al., 1976), or slam freezing – dropping tissue onto a metal block cooled with liquid nitrogen or helium (van Harreveld and Crowell, 1964; Heuser et al., 1979; Heuser and Reese, 1981). Vitreous ice typically forms only within a few micrometers from the surface of the tissue. However, when water is pressurized to 2100 atmospheres, vitreous ice forms more easily and deeply (Kanno et al., 1975; Dahl and Staehelin, 1989; Dubochet, 2007). By applying this level of pressure during rapid freezing, Hans Moore and Udo Riehle obtained good preservation several hundred micrometers from the surface of biological tissues (Riehle, 1968; Moor, 1987).

Frozen samples are then freeze-substituted, a process where the immobilized water is dissolved by an organic solvent (Simpson, 1941). Fixatives such as osmium tetroxide are included in the freeze substitution solvent to fix the sample as it is warmed to room temperature. Once the sample reaches -80°C, secondary ice crystals may grow and disrupt ultrastructure (Steinbrecht, 1985; but see Dubochet, 2007). Thus, organic solvents that are liquid below -80°C, such as acetone, are used for freeze substitution.

The recent availability of commercial high-pressure freezers has made this approach more accessible. Successful high-pressure freezing and freeze-substitution of *C. elegans* preserves ultrastructure and extracellular space better than chemical fixation (Figures 2B,D).

#### High-Pressure Freezing of *C. elegans*

Basic protocols for high-pressure freezing of a range of organisms including *C. elegans* have been described (e.g., Weimer, 2006; McDonald, 2007; Manning and Richmond, 2015). Below is a modified procedure that we have used successfully with both the Leica HPM100 and ICE models of high-pressure freezing machines.

**FIGURE 3:**
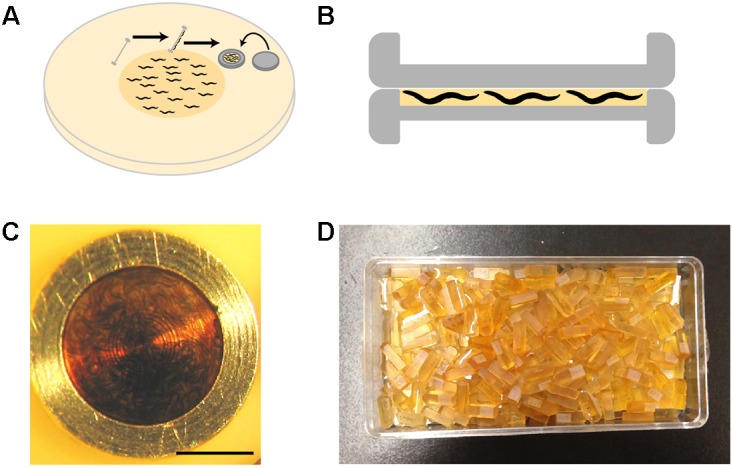
High-pressure freezing of *C. elegans*. **(A)** To pack the carrier with worms, our preferred method is to swipe it across a densely packed lawn of worms and bacteria. After swiping, the worm-bacteria mixture is spread across the cavity of the carrier with tweezers or a worm pick (a thin platinum wire mounted to a holder), the lid put in place, and the sample immediately high-pressure frozen. The entire process takes less than 30 s. **(B)** A carrier when it is packed. It is filled just right, without air bubbles. The smallest cavity for freezing is used, as freezing efficiency decreases with increasing depth. **(C)** A carrier packed with a mixed-staged larva after high-pressure freezing, freeze substitution, and resin infiltration. This carrier has retained the “cake” of worms, but much of the time the cake floats out. One can see how densely the worms are packed by the swiping method. **(D)** Worms are separated from the cake and individually embedded and cured in plastic blocks. Well-packed carriers as shown in panel **(C)** can yield hundreds of intact worm samples.

(a) The carriers in which animals will be frozen (Leica Microsystems, Germany, catalog nos. 16770141 and 16770142) are coated with a non-stick coating (0.1% soy lecithin in chloroform, or 1-hexadecene; McDonald et al., 2010). This coating prevents samples from sticking to the carrier, minimizing damage to samples when they are removed from the carrier.
(b) Worms can be loaded into the 100 μm side of the base carrier using several means (see Tips). The simplest and most effective method is to grow a thick lawn of bacteria and a dense population of worms, and swipe the carrier at an angle of 45° across the surface of the plate to pick up worms with bacteria (Figure 3A). Bacteria act as a filler, minimizing water content and facilitating freezing.
(c) The lid of the carrier is placed on the base immediately prior to freezing (Figure 3B). To preserve animals in their physiological state, we transfer worms from happily eating bacteria on the culture plate to a state of vitreous ice within 30 s.
(d) After freezing, metal carriers that encase frozen samples are transferred under liquid nitrogen into a pre-frozen 1.5 ml cryotube containing 1 ml freeze-substitution solution (see next section), and then to a freeze-substitution unit for processing.

Tips:

• Soy lecithin is an emulsifier that can be obtained economically from baking or health food stores.
• Samples are packed in the 100 μm side of the base carrier because freezing efficiency decreases with increasing depth.
• It is critical that the carrier is completely filled, and there are no air bubbles, which would act as an insulator and also collapse under pressure.
• To freeze samples at defined developmental stages, we either use a synchronized culture, or first fill the carrier with filler, and pick individual animals into the filler. A mixed paste of 10% BSA (dissolved in M9 buffer) and OP50 (an *E. coli* strain commonly used as worm food) forms a nice filler that does not dry up quickly during the loading of individual animals, and allows separation of individual worms after freeze-substitution.
• Samples need to be frozen soon after loading into the carrier to prevent desiccation.
• Some protocols take steps to straighten *C*. *elegans* prior to freezing, either using pharmacological agents (Hall, 1995), or cooling carriers (Bumbarger et al., 2013). We do neither, to eliminate the chance of introducing changes to ultrastructure.

#### Freeze Substitution With *C. elegans* Samples

For morphological analyses, freeze substitution is performed in a programmable freeze substitution unit, where frozen samples are kept at -90°C in the presence of tannic acid and glutaraldehyde, before being replaced by 2% OsO_4_, and brought to room temperature (Box 1; Weimer, 2006). This protocol yields consistent results as long as samples are handled properly (see sections “General Considerations for High-Pressure Freezing and Freeze Substitution” and “High-Pressure Freezing of *C. elegans*”), and the high-pressure freezer is properly assembled and maintained.

**BOX 1:**
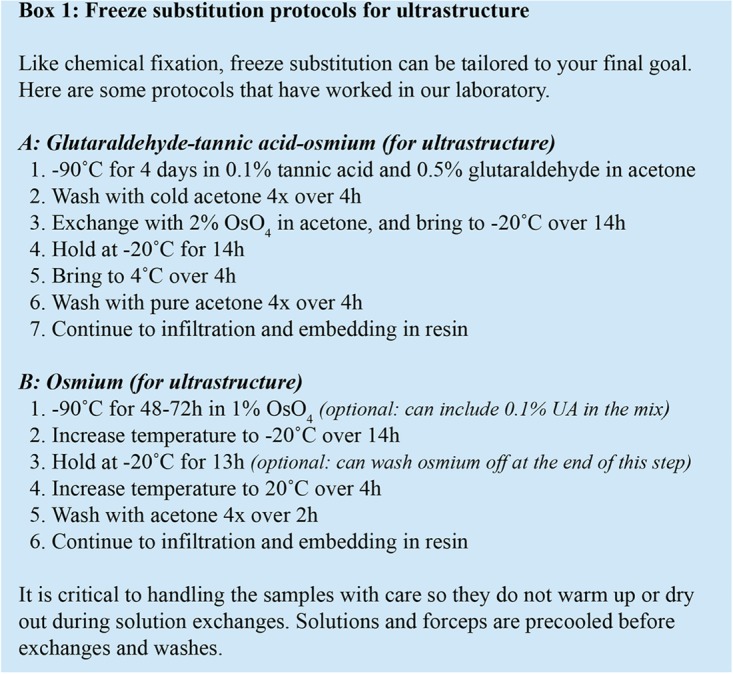
Some freeze substitution protocols for *C. elegans* volume EM. Both **(A)** and **(B)** are effective protocols for ultrastructural preservation (Weimer, 2006).

This protocol can be further modified to reduce processing time and increase the membrane contrast, with the following considerations. Tannic acid helps target osmium to the membrane (Bridgman and Reese, 1984), but glutaraldehyde, inactive at -90°C (Bridgman and Reese, 1984; McDonald, 2007), is likely expendable for the first-step fixation. Inclusion of 5% water in the organic solvent may improve membrane staining (Walther and Ziegler, 2002; Buser and Walther, 2008). To increase heavy metal deposition one can use a mordant to perform a double osmium stain, such as tannic acid (Simionescu and Simionescu, 1976; Wagner, 1976; Jiménez et al., 2009), or thiocarbohydrazide (Seligman et al., 1966; Webb and Schieber, 2018), followed by further *en bloc* uranyl acetate and lead acetate staining (Webb and Schieber, 2018). Lastly, we have confirmed that a fast freeze substitution protocol lasting just a few hours (McDonald and Webb, 2011) also yields well preserved *C. elegans*.

#### Infiltration and Embedding *C. elegans* Samples in Resin

After freeze substitution, the sample needs to be infiltrated with resin and cured in a block. We infiltrate in the same cryotube used for freeze substitution, either in graded steps on a rocker, or employing a fast protocol using centrifugation (McDonald, 2014). For morphology studies carried out by standard TEM and ATUM-SEM, we use Spurr-Quetol resin (NSA 27.88g, ERL4221 9.70g, DER 4.50g, Quetol651 6.12g, and BDMA 0.87g; Ellis, 2006) because it has good sectioning and staining properties, and a relatively low viscosity. For serial block face and FIB-SEM imaging, samples are infiltrated and cured with harder resins, such as hard Epon (EMbed 812 22.6g, DDSA 9.05g, NMA 14.75g, and DMP-30 0.8g) or Durcupan (Durcupan ACM resin 11.4g, DDSA 10.0g, dibutyl phthalate 0.35g, and DMP-30 0.15g).

Once infiltrated, contents of the cryotube are poured into a plate ready for embedding. By this stage, the disk-shaped “cakes” of worms and bacteria will often have fallen out of their carriers. If they are still inside the carrier (Figure 3C), an intact cake can be pried out of the coated carriers using the fine tip of a broken wood stick while holding the carrier in place with tweezers. Using a wooden stick instead of metal instruments is gentler on both the sample and the carriers. We embed either the whole cake, or individual worms released from the cake by repeatedly tapping the cake with the tip of a broken wooden stick until the bacteria crumble away, and intact worms remain (a delicate procedure, especially for young larvae).

Horizontal molds are used to cure samples, as we find it easier to orient samples for subsequent serial sectioning. To place the worm in the center of the block, which makes trimming and cutting easier, we semi-cure half-filled molds by putting them at 60°C for a few hours, let cool, then fill to the top with fresh resin. After we transfer and orient the worms as desired inside the mold, they are cured at 60°C for at least 24 h. The resulting blocks are ready for cutting (Figure 3D).

### Serial Sectioning

Imaging sequential layers of a sample normally requires collecting serial sections for the sample. Although block face imaging techniques avoid this step (Inkson et al., 2001; Denk and Horstmann, 2004; Holzer et al., 2004; Heymann et al., 2006; Knott et al., 2008), samples are destroyed during imaging. There will always be applications for obtaining and preserving long image series. Many effective techniques have been developed (see Box 2).

**BOX 2:**
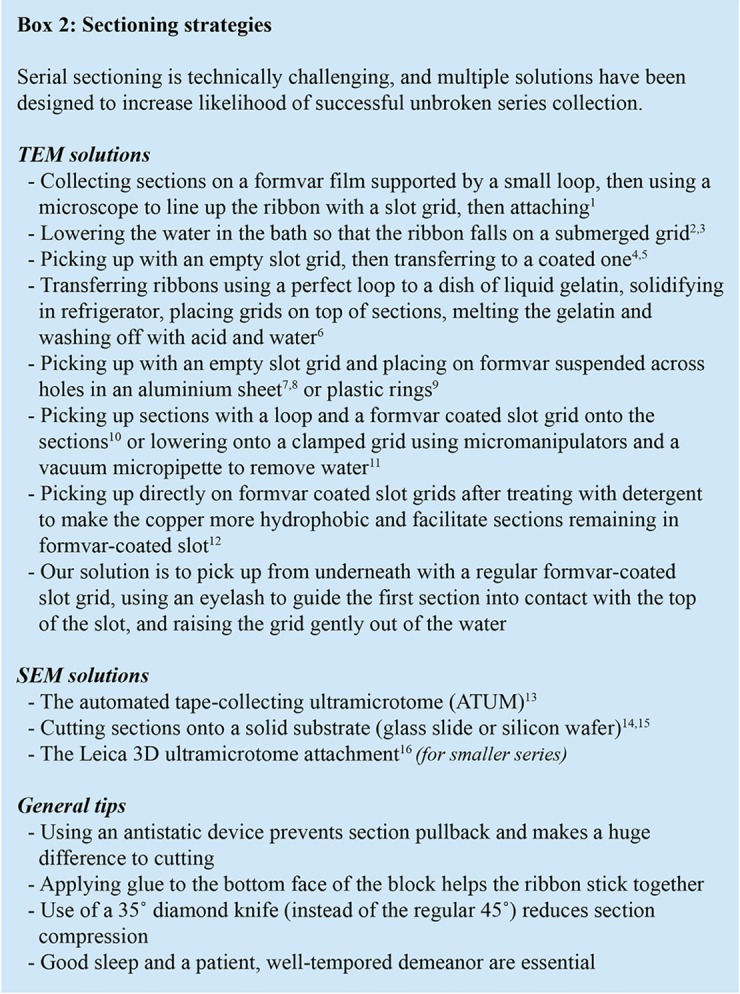
A collection of sectioning strategies for vEM. vEM using non-block face imaging (TEM and SEM) requires collecting large unbroken series of serial sections. There are multiple ways of making the process less error-prone, each with its own merit. One simply has to choose which process works best for them, or devise their own strategy. ^1^*Gay and Anderson (1954);*^2^*Westfall and Healy (1962); ^3^Fahrenbach Wolf (1984);*^4^*Galey and Nilsson (1966); ^5^Mironov et al. (2008);*^6^*Anderson and Brenner (1971);*^7^*Rowley and Moran (1975); ^8^Abad (1988); ^9^Wells (1974); ^10^Mironov et al. (2008); ^11^Stevens et al. (1980); ^12^Hall (1995); ^13^Schalek et al. (2012);*^14^*Micheva and Smith (2007); ^15^Burel et al. (2018); ^16^Leica Microsystems, Germany.*

#### Manual Serial Sectioning for TEM

**FIGURE 4:**
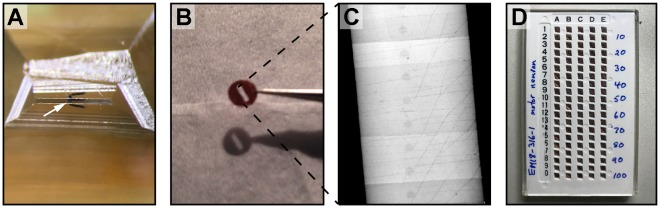
Cutting serial sections for TEM. **(A)** A block face trimmed for cutting. The worm is oriented transversely in the center of the block face (white arrow). **(B)** Ribbons of 10–20 sections are picked up on formvar-coated slot grids. **(C)** A low magnification TEM image of a slot grid, 0.5 mm in diameter. The ribbon of section spans the slot, contributing to the formvar stability. **(D)** Many grids of serial sections, stored in a grid box, are ready for imaging.

(a) Trim the block, leaving a wide surface with the worm in the center (the final block face will be ∼0.7 mm wide).
(b) Collect semi-thin sections when approaching the region of interest using a glass knife. Perform toluidine blue staining to determine the position. Collect ultrathin sections and examine using TEM if precise positioning is necessary.
(c) Once the desired starting position is reached, re-trim the block into a trapezoid with the worm in the center. The height of the trapezoid should be as close to the top and bottom edges of the worm as possible, and the width should be ∼0.7 mm (Figure 4A). Gently dab a thin layer of glue (Elmer’s rubber cement, in a mixture of 1 part glue, 3 parts xylene) to the bottom edge of the block to aid the ribbon formation.
(d) 50 nm serial sections are cut using an ultramicrotome with an antistatic device (we use Static Line Ionizer II, Diatome). Cut as many sections as will fit in the water boat in a single unbroken ribbon. Use a pair of eyelashes glued to wooden sticks to break the long ribbon into smaller ones, which contain 10–20 sections and are able to fit inside a slot grid (Figures 4B,C).
(e) Collect the small ribbons on formvar-coated slot grids. Submerge a grid underneath a ribbon. Hold and align the ribbon with an eyelash, and raise the grid at a 30° angle until the bottom section adheres at the top of the slot. Gently pull up the grid, and the rest of the sections will come with it, with the worm in the center of the slot.
(f) Allow grids to dry before transferring into grid boxes for storage.
(g) Once all sections are picked up, repeat cutting until required volume is complete.
(h) Sections are post-stained with 2% aqueous uranyl acetate and 0.1% lead citrate.

Tips:

• We use 2 mm × 0.5 mm slot grids (instead of 2 mm × 1 mm grids) as there is less chance of damaging the formvar film during handling.
• For serial section datasets, we use commercially prepared 10 nm-thick formvar grids (EMS catalog no. FF205-Cu).
• Make the block face slightly wider than the width of the slot. When the plastic sections span the slot, they contribute to grid stability, reducing the chance of disaster if the formvar is imperfect or becomes damaged (Figure 4).
• Using a 35° diamond knife reduces section compression.
• Holding a stick dipped in xylene or chloroform above the sections corrects compression, but take care not to over-stretch the samples.
• For observing fine details, and tracing neurons that run across the plane of sectioning, 50 nm sections or thinner are necessary.
• The loss of a few sections of a *C. elegans* nerve ring can invalidate the whole dataset for connectome reconstruction. Not only is it difficult to trace through neurons, synapses will also be missing from the final dataset. Handle the grids with care.

#### Automation of Serial Sectioning for SEM

Alternative methods have been devised to automatically cut large volumes of serial sections, including the automated tape collecting ultramicrotome (ATUM; Schalek et al., 2012). Here, the sample is cut on an ultramicrotome and picked up by a rolling reel of tape. The tape is cut into strips, glued to a wafer and post-stained with uranyl acetate and lead citrate. Electrons cannot pass through the tape, therefore scanning electron microscopy (SEM) must be used to image samples cut using an ATUM. We have used this approach to collect serial sections at 30 nm thickness, and used a SEM capable of high resolution imaging (1 nm/pixel; FEI Magellan XHR 400L) to acquire several high-quality datasets for *C. elegans* connectomics studies. Modern high-end SEMs are capable of producing TEM-equivalent micrographs and are suitable for identifying both chemical synapses and gap junctions with high confidence (e.g., Figure 6).

In contrast to the traditional approach of cutting, staining, then imaging sections in an electron microscope, new methods have been established to mount an uncut sample inside the microscope, image the surface using SEM, cut off the top layer, and image again. This process is repeated until the entire region of interest is processed. The cutting uses either a diamond blade inside the microscope (serial block face EM; Denk and Horstmann, 2004), or of a focused ion beam (FIB-SEM; Inkson et al., 2001; Holzer et al., 2004; Heymann et al., 2006; Knott et al., 2008). Both applications can produce images of large volumes for connectomics studies in an exceptionally short amount of time (Briggman and Bock, 2012). Without post-section staining, however, both SBF-SEM and FIB-SEM rely on *en bloc* staining for contrast.

### Image Acquisition and Processing

For connectome reconstruction, we acquired images of entire *C. elegans* cross-sections by either TEM or ATUM-SEM, at 1–2 nm/pixel resolution. We found such a resolution to be necessary for unambiguous annotation of intracellular structures, tracing through small neurites, and synapse annotation. Acquiring the entire cross-section not only allowed us to fully reconstruct dorsal-ventral commissures and lateral nerve cords, but also provided landmarks that facilitated neuron identification.

After sections are imaged, they are stitched and aligned into a 3D volume. This requires processing of acquired images to compensate for artifacts generated during sectioning (e.g., differential compression of sections), and imaging (e.g., lens distortion, shrinkage of samples due to the energy of the electron beam). There are multiple solutions for alignment of datasets into 3D volumes (reviewed in Borrett and Hughes, 2016). We found TrakEM2 (Saalfeld et al., 2010; Cardona et al., 2012) to be most suitable for our *C. elegans* datasets, and we outline the process below.

(a) Sections are imaged at the required resolution in the electron microscope. Imaging at a resolution of 1–2 nm per pixel is optimal for tracing fine processes and mapping small synapse with high confidence.
(b) When a region of interest does not fit into the field of view of the camera, it is imaged as a montage with 10% overlap on each side.
(c) A text file is generated containing the paths to the images and their respective coordinates in x, y, and z, then used to import the dataset into TrakEM2.
(d) Once the dataset is imported into TrakEM2, image filters are applied to optimize brightness and contrast throughout the dataset.
(e) The lens correction function in TrakEM2 is used to correct for lens distortion caused by imperfect lenses in the electron microscope. Using a set of heavily overlapping images, the distortion of images is calculated, and a correction is applied to each image in the dataset.
(f) Each section is montaged rigidly in x-y using the TrakEM2 least-squares alignment tool.
(g) Each section is montaged elastically in x-y using the TrakEM2 elastic alignment tool.
(h) Layers are aligned rigidly in z using the TrakEM2 least-squares alignment tool.
(i) Layers are aligned elastically in z using the TrakEM2 elastic alignment tool.
(j) Images are exported from TrakEM2 either as flat images, or tiles ready for importing into an instance of CATMAID.

Tips:

• Samples on slot grids shrink when exposed to the electron beam. We reduce the shrinkage by coating these grids with a thin layer of carbon, and “prebaking” each section at a lower magnification in the electron beam for around 1 min before imaging.
• Automatic montaging is a function available in some camera softwares (e.g., Gatan Microscopy Suite). Free software such as SerialEM is capable of performing montages and compatible with a range of cameras (Mastronarde, 2005).
• Text files with paths to the images and coordinates can be generated in various ways. We use a Python script to extract the paths from the folder containing the images, and set the coordinates. It can also be done manually in Excel. Consistent file naming and number padding facilitate this step.
• Adjustable parameters for stitching are numerous and daunting. The TrakEM2 manual^1^ and ImageJ feature extraction page^2^ provide guides for parameter selection. Optimal parameters for each dataset have to be worked out through trial and error. Test a few sections at a time until all images can be reasonably well aligned.
• Manual inspection and correction is necessary for each step. We frequently use the transform function while superimposing a transparent copy of the previous layer to register poorly aligned sections. Using manually placed landmarks to register multiple sections is also an effective strategy.
• Care must be taken not to distort or twist the images whilst proceeding through the image stack.

### Segmentation

We have used several open-source software packages for manual segmentation of image stacks. For small image stacks, we have used Reconstruct (Fiala, 2005; Yeh et al., 2009; Hung et al., 2013) and TrakEM2 (Cardona et al., 2012; Meng et al., 2015; Lim et al., 2016) for volumetric reconstruction. For connectomics studies, which requires handling of large image datasets, we have used CATMAID (collaborative annotation toolkit for massive amounts of imaging data; Saalfeld et al., 2009) for skeleton tracing, and VAST (Volume Annotation and Segmentation Tool; Kasthuri et al., 2015) for volume reconstruction.

#### Skeleton Tracing With CATMAID

To generate *C. elegans* connectomes, we apply skeleton tracing to reconstruct all neurons and their connectivity. Skeleton tracing consists of placing dots, or “nodes,” in the center of a neurite throughout the volume, forming a skeleton as the tracing progresses. Compared to volumetric reconstruction, skeleton tracing allows faster manual reconstruction of the nervous system. With a high-quality dataset, a first larval stage nerve ring (the worm central nervous system) can be manually traced to completion by a well-trained and committed tracer in a few days. As neurons are traced, they are identified based on stereotypic structures and connectivity patterns, along with neurite trajectory and placement, and cell body position (see below). Ambiguities may arise due to artifacts such as section folding or stain precipitation, and can be resolved by completing the tracing of the rest of the neurons in the immediate area. Neurons are identifiable by features distributed throughout the nerve ring.

**FIGURE 5:**
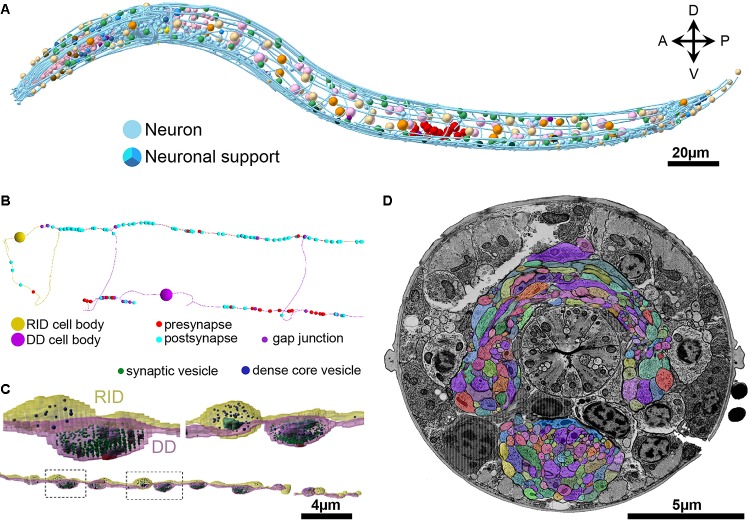
Skeleton and volumetric reconstruction of the *C. elegans* nervous system. **(A)** A complete reconstruction of all nuclei (round balls) and all neuronal processes (blue cables) of a first larval stage *C. elegans*, achieved through skeleton tracing in CATMAID, and visualized with Blender. **(B)** A skeleton reconstruction of anterior DD-type motor neurons and the neuromodulatory neuron RID generated using CATMAID. Synaptic input and output are indicated by cyan and red spheres, respectively, and putative gap junctions in marked in dark purple. **(C)** Volumetric segmentation of part of a DD motor neuron and RID using TrakEM2, with intracellular ultrastructure segmented. **(D)** A cross-section of an L1 larva. Its nerve ring was fully reconstructed by volumetric segmentation. These segmentation profiles were generated by expanding skeleton seeds to a membrane probability map, followed by manual proofreading in VAST.

After neurite tracing is complete, connectors can be placed between nodes of different skeletons to signify chemical synapses and gap junctions. Visualization of neuron skeletons in 3D is often sufficient for assessing the coarse position and process trajectory of individual neurons, as well as the overall architecture of neuropils and ganglia (Figures 5A,B). However, substantial morphological information is omitted.

#### Volumetric Segmentation With VAST

To accurately obtain morphological information such as neuron size, shape, and the relative contact area between neurons, volumetric segmentation is necessary. Additional segmentation of intracellular ultrastructure can yield information such as the distribution, morphology, number, and size of microtubules, mitochondria, ER, presynaptic densities, synaptic and dense core vesicles and other vesicular structures. This is useful to understand the cell biology of the neuron (Figure 5C).

The VAST software package is capable of segmenting in such a way (Kasthuri et al., 2015). In our hands, VAST has the best performance when handling large datasets like the entire *C. elegans* nerve ring (Figure 5D). Manual volumetric segmentation, however, is very low throughput. Fully automated segmentation methods have been reported, but they have yet to perform well with our *C. elegans* datasets. We took an alternative, semi-automated approach. In this approach, membrane probability maps were generated from small training stacks (Meirovitch et al., 2016), and nodes that were generated from skeleton tracing were expanded to the calculated membrane boundary to fill the neurite (Meirovitch et al., in preparation). This is followed by manual proof-reading in VAST (Figure 5D).

### Synapse Annotation

Different fixation protocols can lead to differences in the morphology of fixed tissues. Therefore, it is important to adjust criteria for synapse annotation for datasets generated using different fixation protocols and imaging conditions. For example, the slow fixation protocol used for generating the original *C. elegans* adult wiring datasets was optimized for cell membrane contrast. Fine intracellular ultrastructure was less well preserved, and presynaptic dense projections appear as a dark density close to the membrane, with hard to discern morphology. This makes chemical synapse annotation more prone to staining artifacts. The slow fixation protocol caused shrinkage of neurites, which tore apart weak adhesions between adjacent neurites. Such a distortion could complicate the assignment of postsynaptic partners in polyadic synapses, but highlight gap junctions, which remain intact. Synapse annotation and connectome assembly were carried out cautiously and carefully with these caveats in mind (White et al., 1986). Any reconsideration of these micrographs should involve careful study of the entire dataset and apply similarly rigorous criteria to avoid the “false positive” identification of synapses.

**FIGURE 6:**
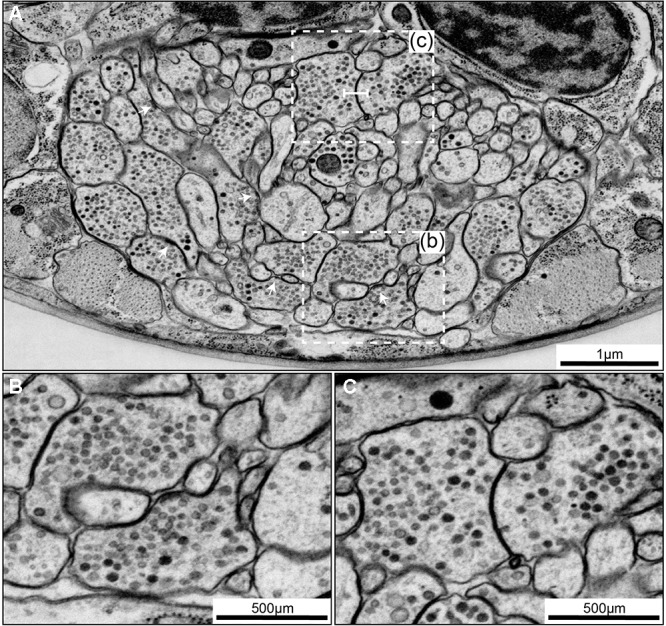
Chemical synapses and gap junctions in *C. elegans*. **(A)** A section of the first larva (L1) ventral ganglion neuropil imaged using SEM at 1 nm/pixel. Multiple chemical synapses are visible (white arrows) as well as a gap junction (white flat-ended line). **(B)** Enlarged view of the chemical synapse highlighted with a dashed box in panel **(A)**. There is a presynaptic dense projection and a pool of synaptic vesicles, as well as some dense core vesicles further back in the neurite. This synapse is polyadic, releasing onto three neurons. **(C)** Enlarged view of the gap junction highlighted with a dashed box in panel **(A)**. There is a relatively flat area of close apposition between the membranes.

**FIGURE 7:**
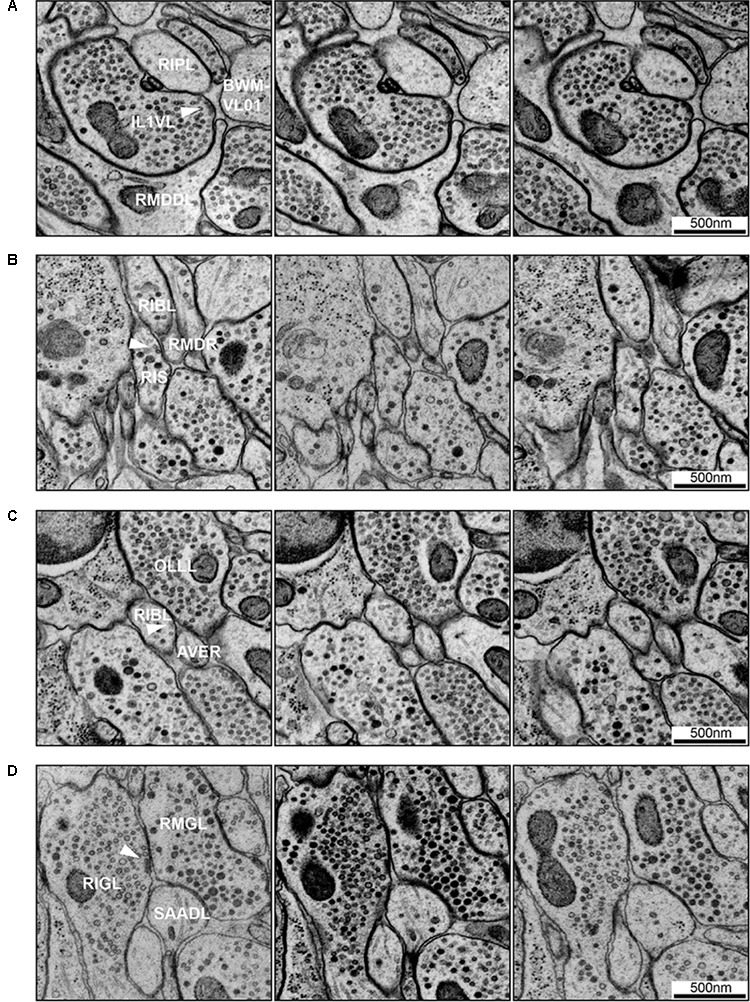
Examples of synapse annotation with different degrees of subjectivity. **(A)** Serial sections through a large, confidently annotated polyadic synapse (from IL1VL to RIPL, RMDDL and body wall muscle BWM-VL01). This synapse spans these three sections, and beyond (not shown). **(B)** Serial sections through a very small synapse (from RIS to RIBL and RMDR). The annotation of this synapse is less confident that the one presented in panel **(A)**. **(C)** Serial sections of a membrane swelling that is confidently annotated as not-a-synapse. A small density in the membrane of RIBL with sparse vesicles is not a presynaptic specialization. **(D)** Serial sections through a synapse showing the occasional subjectivity involved in defining postsynaptic partners. While all annotators agreed RMGL was a postsynaptic partner of RIGL, whether SAADL should be included as a postsynaptic partner was cause for debate. White arrowheads indicate the membrane of interest. Scale bars are 500 μm.

Even with a well-preserved sample that has been fixed using high-pressure freezing and aligned well into a 3D volume, synapse annotation requires training, and includes of element of subjectivity (see below; Figures 6, 7). For a compact nervous system such as *C. elegans*, where neuron and synapse numbers are small, it is even more pertinent to establish stringent criteria for sample preparation and synapse annotation, and to obtain and compare multiple datasets from isogenic individuals, so that errors can be minimized.

Below we describe the criteria used for synapse annotation in our high-pressure frozen and freeze substituted volumes of the *C. elegans* nervous system.

#### Chemical Synapses

*Caenorhabditis elegans* presynapses generally consist of a swelling in the neurite, with a visible electron-dense presynaptic density attached to the plasma membrane marking the active zone, with a cloud of vesicles adjacent to the presynaptic density (Figures 6A,B, 7A). Vesicle clouds often consist of many clear core synaptic vesicles close to the active zone, and a small number of large, dense-core vesicles that reside more peripherally. Vesicle clouds can cover large areas with multiple small presynaptic dense projections, especially in the nerve ring. If the synapse is small, cut at an awkward angle, or if there are artifacts covering or interfering with the putative synapse, assigning whether it is a synapse or not can sometimes be a bit subjective (Figures 7B,C). Many synapses are polyadic. Since most synapses in the *C. elegans* nervous system do not have visible postsynaptic densities, postsynaptic partners are assigned based on their proximity to the presynaptic active zones, which can be a source of subjectivity (Figure 7D).

To minimize the problem of subjectivity, our datasets are fully annotated by three independent annotators. Using CATMAID one can assign confidence scores to synapses, with a score of 5 indicating a high level of confidence, and a score of 1 indicating very low confidence. The triplicate annotations are then merged, and every inconsistency between annotators is flagged for discussion. If agreement is not reached by the three annotators after debate, an average of the confidence scores is reported to allow subsequent data users to make their own judgments.

#### Gap Junctions

Gap junctions are notoriously difficult to identify in vEM. There are some morphological criteria that can help identify some with reasonable certainty. A classic gap junction profile includes a close, relatively flat area of membrane apposition of limited extracellular space (∼2 nm) across multiple sections, a thicker membrane, with a characteristic sharp zippering of the membranes immediately at the boundaries of the putative gap junction (Figure 6C). These features can be quite clear if cut at the perfect angle with thin (30–50 nm) sections, but even in well-stained samples not all gap junctions can be marked unambiguously. Tomography, which acquires images of the same section at different tilt angles to generate a high-resolution 3D volume of the section, helps survey a putative gap junction, but it is unrealistic to apply such an approach to the entire series of the nervous system.

We corroborate our gap junction annotation by comparing patterns across our multiple new datasets and to the original datasets (White et al., 1976, 1986). The slow chemical fixation protocol used for the original adult connectome, while distorting neurite morphology and pulling apart weaker contacts between neurites, allowed strong membrane connections such as gap junctions to be particularly well distinguished. Some of the morphologically identified gap junctions have been functionally validated (Chalfie et al., 1985; Liu et al., 2017). Comparing new and old datasets allows us to refine criteria for gap junction annotation in high-pressure frozen datasets. These criteria are validated by uncovering recurrent gap junction-like structures when comparing the same membranes between neuronal classes across datasets. Because in each sample, the junction between each neuron pair was sectioned from a different angle, stereotypic gap junctions can be confirmed in multiple views. Our approach will likely miss small or sparse gap junctions.

Multiple approaches have been attempted to highlight gap junctions in EM volumes. CLEM (correlative light and electron microscopy), where gap junctions are labeled by immunostaining against one of the *C. elegans* innexin::GFP fusions, showed promise (Markert et al., 2016, 2017). This approach requires a weak fixation that compromises structural preservation, and it would be difficult to expand this approach to all 25 *C. elegans* innexins. We and others are working to develop EM preservation protocols to improve gap junction annotation.

### Neuron Identification

In a large, good quality *C. elegans* volume, every single cell can be assigned its unique cell name. Each neuron class has been described in such superb detail in *The Mind of a Worm* (White et al., 1986) that by reading the neuron descriptions while going through the complete EM series, one can identify neurons one by one throughout the volume. *WormAtlas* hosts scanned copies of the neuron pages from *The Mind of a Worm* that are accessible through a drop-down menu in an internet browser (Altun et al., 2002–2018). Several features indicate neuron identity: cell body position, neurite trajectory, stereotypic neurite placement or morphology and stereotypic connectivity patterns. We found that this stereotypy holds across postnatal developmental stages for most neurons, with a few exceptions.

**FIGURE 8:**
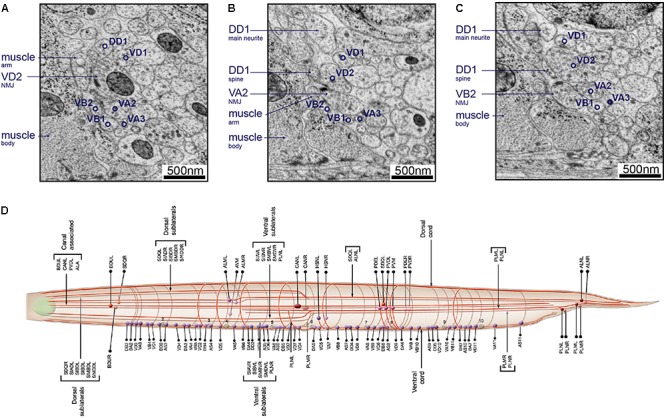
Neurons can be identified from 3D volumes. Electron micrographs showing snapshots of part of the ventral nerve cord from an animal at the end of the second larval stage, imaged using SEM at 2 nm/pixel. **(A)** A VD2 NMJ is pointing laterally toward a muscle arm. This example also “hits” a projection from the VA2 motor neuron, but it is not clear if receptors are present. Some other motor neurons are also labeled, to give a sense of the relative position within the nerve cord. **(B)** A VA2 NMJ is pointing more dorsally, releasing onto a muscle arm, a DD1 spine and VD2. **(C)** A VB2 NMJ is also pointed dorsally, releasing onto muscle, a DD1 spine and VD2. **(D)** A cartoon of most of the commissure bundles in *C. elegans*, available on *WormAtlas* (Altun et al., 2002–2018) and based on The Mind of a Worm (White et al., 1986). The positions, handedness and commissure bundle partners are known, and very stereotypic. Bundles of neuron processes are shown as red cables. The cell bodies are denoted with spheres, and also have stereotypic positions along the body of the worm and relative to each other.

For example, in the adult ventral nerve cord, VC processes are generally most dorsal, followed by VD, DD, VA, then VB toward the ventral side. Synapses to body wall muscles come from VA, VB, VD, and VC class motor neurons. Among them, VD presynaptic swellings are large, face directly toward the muscle, most of the time without any neurons as dyadic postsynaptic partners (Jin et al., 1999; White et al., 1976, 1986; Figure 8A). On the other hand, VA and VB, form NMJs that consist of smaller swellings, are often on the dorsal side of the neurite, and almost always dyadic with DD dendrites, which send spine-like structure toward the NMJ (White et al., 1976, 1986; Jin et al., 1999; White, 2013; Figures 8B,C).

Neurite trajectory and process placement are used to further identify neurons. For example, VAs project axons anteriorly from the soma, whereas VB axons project posteriorly. VDs also project their axons anteriorly, but they send a dorsal-projecting commissure at the end of the axon regions. Commissure trajectory (whether it exits the ventral nerve cord from the left or right side) and partners in each commissure bundle further assist cell identification (Figure 8D). For example, VD2 runs in a left-handed commissure, always bundled with that of DD1, DA1, and DB2.

These, and other observations, allow one to recognize the “fingerprints” of motor neuron identity. Similar observations and strategies apply to the other neuropils in the worm, such as the dorsal nerve cord, the nerve ring, and the other cords and ganglia of the worm, as well as across different stages of development. Some neurons are not born until later in development (Sulston and Horvitz, 1977), but most neurons have stereotypic features and connectivity across larval stages. A notable exception is the DD motor neuron class, which exhibits extensive remodeling of connectivity during development (White et al., 1978).

### Assembly of a Wiring Matrix

After obtaining a connectome, we further assess pairwise connections to gauge confidence in biologically relevant connections. Connections between two neurons consisting of many synapses are considered high confidence. A connection is considered uncertain if it consists of very few synapses. When few synapses are observed between neurons, we often observe inconsistency in the existence of the connection across animals. From comparing multiple datasets that we have acquired for the *C. elegans* nerve ring and ventral ganglion, three synapses seem to be a sensible lower bound on a high confidence connection. Even so, to minimize variability introduced by annotators, and assess true biological variability, acquiring connectomes from multiple animals is advisable.

## Perspectives

The pipeline described above represents only a starting point for modern high throughput *C. elegans* vEM. We should expect rapid and substantial improvement both in terms of throughput and quality. Future improvements will include automated image segmentation, synapse annotation and neuron and neurite identification. This will be facilitated by the generation of new *C. elegans* connectomes as training datasets for machine learning approaches. Incorporating of these improvements will allow not only rapid reconstruction of connectomes from multiple animals, but also facilitate targeted reconstruction of specific segments of the nervous system by computer vision.

The *C. elegans* nervous system is compact, allowing precise correlation of anatomy (connectome) with membrane physiology (activity and excitability of individual neurons), sign of synaptic communication (neurotransmitter and receptor of individual synapses), and behavior. The delineation of the neurotransmitter type and receptor complement of each neuron (Serrano-Saiz et al., 2013; Pereira et al., 2015; Gendrel et al., 2016), combined with the connectivity, allow for more sophisticated modeling of information flow through the nervous system. Whole brain calcium imaging from fixed and behaving animals allows observation of the activity of functioning neural circuitry (Schrödel et al., 2013; Prevedel et al., 2014; Kato et al., 2015; Nguyen et al., 2016; Venkatachalam et al., 2016), allowing correlation of anatomic and functional connectivity. Performing connectomics on animals with genetic mutations that affect diverse properties of neurons – neuronal fate, synaptic transmission, cell adhesion and signaling – holds the promise of identifying genetic and biochemical pathways that determine connectivity. This system holds a promise to reveal insight on principles of how a connectome leads to hard-wired and flexible behaviors (Johnson et al., 1995; Harris-Warrick et al., 1998; Marder and Bucher, 2007; Agnati et al., 2010).

The field of *C. elegans* connectomics is at a new beginning. Modern techniques now allow us to use connectomics to address questions about the dynamic and comparative structures of complete nervous systems. How does a connectome remodel across development? What sexual dimorphisms are held within a connectome? How do mutations in genes that establish the trajectory of neurite growth, the specificity of synapse partners, and the molecular complement of the plasma membrane, change a connectome? Does a connectome drift with age? How much inter-individual variability is there? Is learning and memory physically manifested within the connectome? What about the influence of environment? How are the behavioral differences between morphologically similar but evolutionarily distinct *Caenorhabditis* species represented by the connectome? How does a connectome evolve?

Finally, volume EM of *C. elegans* does not only generate information about the nervous system. Packaged within the small volume, our volumes of the nervous system data also capture other tissues – the skin, gut, musculature, excretory cells, and reproductive system – each with their own exquisite intracellular ultrastructure. All datasets will be useful to the much larger community of biologists.

## Author Contributions

All authors contributed to developing the approaches described above, as well as writing and editing the manuscript.

## Conflict of Interest Statement

The authors declare that the research was conducted in the absence of any commercial or financial relationships that could be construed as a potential conflict of interest.

## Footnotes

1 www.ini.uzh.ch/acardona/trakem2_manual.html

2 http://imagej.net/Feature_Extraction

## References

[B1] Abad A. (1988). A study of section wrinkling on single-hole coated grids using TEM and SEM. *J. Electron Microsc. Tech.* 8 217–222. 10.1002/jemt.1060080209 3246609

[B2] Agnati L. F. Guidolin D. Guescini M. Genedani S. Fuxe K. (2010). Understanding wiring and volume transmission. *Brain Res. Rev.* 64 137–159. 10.1016/j.brainresrev.2010.03.003 20347870

[B3] Albertson D. G. Thomson J. N. (1976). The pharynx of *Caenorhabditis elegans*. *Philos. Trans. R. Soc. Lond. B Biol. Sci.* 275 299–325. 10.1098/rstb.1976.0085 8805

[B4] Allen E. Ren J. Zhang Y. Alcedo J. (2015). Sensory systems: their impact on *C. elegans* survival. *Neuroscience* 296 15–25. 10.1016/j.neuroscience.2014.06.054 24997267 PMC4282626

[B5] Altun Z. F. Herndon L. A. Wolkow C. A. Crocker C. Lints R. Hall D. H. (2002–2018). *WormAtlas.* Available at: http://www.wormatlas.org.

[B6] Anderson R. G. W. Brenner R. M. (1971). Accurate placement of ultrathin sections on grids; control by sol-gel phases of a gelatin flotation fluid. *Stain Technol.* 46 1–6. 10.3109/10520297109067809 5542103

[B7] Ardiel E. L. Rankin C. H. (2010). An elegant mind: learning and memory in *Caenorhabditis elegans*. *Learn. Mem.* 17 191–201. 10.1101/lm.960510 20335372

[B8] Boergens K. M. Berning M. Bocklisch T. Bräunlein D. Drawitsch F. Frohnhofen J. (2017). webKnossos: efficient online 3D data annotation for connectomics. *Nat. Methods* 14 691–694. 10.1038/nmeth.4331 28604722

[B9] Borrett S. Hughes L. (2016). Reporting methods for processing and analysis of data from serial block face scanning electron microscopy. *J. Microsc.* 263 3–9. 10.1111/jmi.12377 26800017

[B10] Bridgman P. C. Reese T. S. (1984). The structure of cytoplasm in directly frozen cultured cells. I. Filamentous meshworks and the cytoplasmic ground substance. *J. Cell Biol.* 99 1655–1668. 10.1083/jcb.99.5.1655 6436253 PMC2113346

[B11] Briggman K. L. Bock D. D. (2012). Volume electron microscopy for neuronal circuit reconstruction. *Curr. Opin. Neurobiol.* 22 154–161. 10.1016/j.conb.2011.10.022 22119321

[B12] Bumbarger D. J. Riebesell M. Rödelsperger C. Sommer R. J. (2013). System-wide rewiring underlies behavioral differences in predatory and bacterial-feeding nematodes. *Cell* 152 109–119. 10.1016/j.cell.2012.12.013 23332749

[B13] Burel A. Lavault M.-T. Chevalier C. Gnaegi H. Prigent S. Mucciolo A. (2018). A targeted 3D EM and correlative microscopy method using SEM array tomography. *Development* 145:dev160879. 10.1242/dev.160879 29802150

[B14] Buser C. Walther P. (2008). Freeze-substitution: the addition of water to polar solvents enhances the retention of structure and acts at temperatures around –60°C. *J. Microsc.* 230 268–277. 10.1111/j.1365-2818.2008.01984.x 18445157

[B15] Cardona A. Saalfeld S. Schindelin J. Arganda-Carreras I. Preibisch S. Longair M. (2012). TrakEM2 software for neural circuit reconstruction. *PLoS One* 7:e38011. 10.1371/journal.pone.0038011 22723842 PMC3378562

[B16] Chalfie M. Sulston J. E. White J. G. Southgate E. Thomson J. N. Brenner S. (1985). The neural circuit for touch sensitivity in *Caenorhabditis elegans*. *J. Neurosci.* 5 956–964. 10.1523/JNEUROSCI.05-04-00956.1985 3981252 PMC6565008

[B17] Dahl R. Staehelin L. A. (1989). High-pressure freezing for the preservation of biological structure: theory and practice. *J. Electron Microsc. Tech.* 13 165–174. 10.1002/jemt.1060130305 2685196

[B18] Denk W. Horstmann H. (2004). Serial block-face scanning electron microscopy to reconstruct three-dimensional tissue nanostructure. *PLoS Biol.* 2:e329. 10.1371/journal.pbio.0020329 15514700 PMC524270

[B19] Dubochet J. (2007). The physics of rapid cooling and its implications for cryoimmobilization of cells. *Methods Cell Biol.* 79 7–21. 10.1016/S0091-679X(06)79001-X 17327150

[B20] Durbin R. M. (1987). *Studies on the Development and Organisation of the Nervous System of Caenorhabditis elegans.* Ph.D. thesis, Cambridge, MRC Laboratory of Molecular Biology.

[B21] Eichler K. Li F. Litwin-Kumar A. Park Y. Andrade I. Schneider-Mizell C. M. (2017). The complete connectome of a learning and memory centre in an insect brain. *Nature* 548 175–182. 10.1038/nature23455 28796202 PMC5806122

[B22] Ellis A. E. (2006). Solutions to the problem of substitution of ERL 4221 for vinyl cyclohexene dioxide in spurr low viscosity embedding formulations. *Micros. Today* 14 32–33. 10.1017/S1551929500050252

[B23] Fahrenbach Wolf H. (1984). Continuous serial thin sectioning for electron microscopy. *J. Electron Microsc. Tech.* 1 387–398. 10.1002/jemt.1060010407

[B24] Feder N. Sidman R. L. (1958). Methods and principles of fixation by freeze-substitution. *J. Biophys. Biochem. Cytol.* 4 593–602. 10.1083/jcb.4.5.593 13587555 PMC2224544

[B25] Fiala J. C. (2005). Reconstruct: a free editor for serial section microscopy. *J. Microsc.* 218 52–61. 10.1111/j.1365-2818.2005.01466.x 15817063

[B26] Galey F. R. Nilsson S. E. G. (1966). A new method for transferring sections from the liquid surface of the trough through staining solutions to the supporting film of a grid. *J. Ultrastruct. Res.* 14 405–410. 10.1016/S0022-5320(66)80057-6 4160139

[B27] Gay H. Anderson T. F. (1954). Serial sections for electron microscopy. *Science* 120 1071–1073. 10.1126/science.120.3130.1071 13216224

[B28] Gendrel M. Atlas E. G. Hobert O. (2016). A cellular and regulatory map of the GABAergic nervous system of *C. elegans*. *eLife* 5:e17686. 10.7554/eLife.17686 27740909 PMC5065314

[B29] Gilkey J. C. Staehelin L. A. (1986). Advances in ultrarapid freezing for the preservation of cellular ultrastructure. *J. Electron Microsc. Tech.* 3 177–210. 10.1002/jemt.1060030206

[B30] Hall D. H. (1995). “Electron microscopy and three-dimensional image reconstruction,” in *Methods in Cell Biology*, eds Epstein H. F. Shakes D. C. (Cambridge, MA: Academic Press), 395–436. 10.1016/s0091-679x(08)61397-7 8531736

[B31] Hall D. H. Russell R. L. (1991). The posterior nervous system of the nematode *Caenorhabditis elegans*: serial reconstruction of identified neurons and complete pattern of synaptic interactions. *J. Neurosci.* 11 1–22. 10.1523/JNEUROSCI.11-01-00001.1991 1986064 PMC6575198

[B32] Harris-Warrick R. M. Johnson B. R. Peck J. H. Kloppenburg P. Ayali A. Skarbinski J. (1998). Distributed effects of dopamine modulation in the crustacean pyloric network. *Ann. N. Y. Acad. Sci.* 860 155–167. 10.1111/j.1749-6632.1998.tb09046.x 9928309

[B33] Hayworth K. J. Morgan J. L. Schalek R. Berger D. R. Hildebrand D. G. C. Lichtman J. W. (2014). Imaging ATUM ultrathin section libraries with WaferMapper: a multi-scale approach to EM reconstruction of neural circuits. *Front. Neural Circuits* 8:68. 10.3389/fncir.2014.00068 25018701 PMC4073626

[B34] Helmstaedter M. Briggman K. L. Denk W. (2011). High-accuracy neurite reconstruction for high-throughput neuroanatomy. *Nat. Neurosci.* 14 1081–1088. 10.1038/nn.2868 21743472

[B35] Helmstaedter M. Briggman K. L. Turaga S. C. Jain V. Seung H. S. Denk W. (2013). Connectomic reconstruction of the inner plexiform layer in the mouse retina. *Nature* 500 168–174. 10.1038/nature12346 23925239

[B36] Heuser J. E. Reese T. S. (1981). Structural changes after transmitter release at the frog neuromuscular junction. *J. Cell Biol.* 88 564–580. 10.1083/jcb.88.3.564 6260814 PMC2112753

[B37] Heuser J. E. Reese T. S. Dennis M. J. Jan Y. Jan L. Evans L. (1979). Synaptic vesicle exocytosis captured by quick freezing and correlated with quantal transmitter release. *J. Cell Biol.* 81 275–300. 10.1083/jcb.81.2.275 38256 PMC2110310

[B38] Heymann J. A. W. Hayles M. Gestmann I. Giannuzzi L. A. Lich B. Subramaniam S. (2006). Site-specific 3D imaging of cells and tissues with a dual beam microscope. *J. Struct. Biol.* 155 63–73. 10.1016/j.jsb.2006.03.006 16713294 PMC1647295

[B39] Holzer L. Indutnyi F. Gasser P. Münch B. Wegmann M. (2004). Three-dimensional analysis of porous BaTiO3 ceramics using FIB nanotomography. *J. Microsc.* 216 84–95. 10.1111/j.0022-2720.2004.01397.x 15369488

[B40] Hung W. L. Hwang C. Gao S. Liao E. H. Chitturi J. Wang Y. (2013). Attenuation of insulin signalling contributes to FSN-1-mediated regulation of synapse development. *EMBO J.* 32 1745–1760. 10.1038/emboj.2013.91 23665919 PMC3680742

[B41] Inkson B. J. Mulvihill M. Möbus G. (2001). 3D determination of grain shape in a FeAl-based nanocomposite by 3D FIB tomography. *Scr. Mater.* 45 753–758. 10.1016/S1359-6462(01)01090-9

[B42] Jarrell T. A. Wang Y. Bloniarz A. E. Brittin C. A. Xu M. Thomson J. N. (2012). The connectome of a decision-making neural network. *Science* 337 437–444. 10.1126/science.1221762 22837521

[B43] Jiménez N. Vocking K. van Donselaar E. G. Humbel B. M. Post J. A. Verkleij A. J. (2009). Tannic acid-mediated osmium impregnation after freeze-substitution: a strategy to enhance membrane contrast for electron tomography. *J. Struct. Biol.* 166 103–106. 10.1016/j.jsb.2008.12.009 19162195

[B44] Jin Y. Jorgensen E. Hartwieg E. Horvitz H. R. (1999). The *Caenorhabditis elegans* Gene *unc-25* encodes glutamic acid decarboxylase and is required for synaptic transmission but not synaptic development. *J. Neurosci.* 19 539–548. 10.1523/JNEUROSCI.19-02-00539.1999 9880574 PMC6782196

[B45] Johnson B. R. Peck J. H. Harris-Warrick R. M. (1995). Distributed amine modulation of graded chemical transmission in the pyloric network of the lobster stomatogastric ganglion. *J. Neurophysiol.* 74 437–452. 10.1152/jn.1995.74.1.437 7472345

[B46] Kanno H. Speedy R. J. Angell C. A. (1975). Supercooling of water to -92°C under pressure. *Science* 189:880. 10.1126/science.189.4206.880 17812529

[B47] Kasthuri N. Hayworth K. J. Berger D. R. Schalek R. L. Conchello J. A. Knowles-Barley S. (2015). Saturated reconstruction of a volume of neocortex. *Cell* 162 648–661. 10.1016/j.cell.2015.06.054 26232230

[B48] Kato S. Kaplan H. S. Schrodel T. Skora S. Lindsay T. H. Yemini E. (2015). Global brain dynamics embed the motor command sequence of *Caenorhabditis elegans*. *Cell* 163 656–669. 10.1016/j.cell.2015.09.034 26478179

[B49] Knott G. Marchman H. Wall D. Lich B. (2008). Serial section scanning electron microscopy of adult brain tissue using focused ion beam milling. *J. Neurosci.* 28 2959–2964. 10.1523/JNEUROSCI.3189-07.2008 18353998 PMC6670719

[B50] Lim M. A. Chitturi J. Laskova V. Meng J. Findeis D. Wiekenberg A. (2016). Neuroendocrine modulation sustains the *C. elegans* forward motor state. *eLife* 5:e19887. 10.7554/eLife.19887 27855782 PMC5120884

[B51] Liu P. Chen B. Mailler R. Wang Z.-W. (2017). Antidromic-rectifying gap junctions amplify chemical transmission at functionally mixed electrical-chemical synapses. *Nat. Commun.* 8:14818. 10.1038/ncomms14818 28317880 PMC5364397

[B52] Manning L. Richmond J. (2015). “High-pressure freeze and freeze substitution electron microscopy in *C. elegans*,” in *C. elegans: Methods and Applications*, eds Biron D. Haspel G. (Totowa, NJ: Humana Press), 121–140. 10.1007/978-1-4939-2842-2_10 26423972

[B53] Marder E. Bucher D. (2007). Understanding circuit dynamics using the stomatogastric nervous system of lobsters and crabs. *Annu. Rev. Physiol.* 69 291–316. 10.1146/annurev.physiol.69.031905.161516 17009928

[B54] Markert S. M. Bauer V. Muenz T. S. Jones N. G. Helmprobst F. Britz S. (2017). 3D subcellular localization with superresolution array tomography on ultrathin sections of various species. *Methods Cell Biol.* 140 21–47. 10.1016/bs.mcb.2017.03.004 28528634

[B55] Markert S. M. Britz S. Proppert S. Lang M. Witvliet D. Mulcahy B. (2016). Filling the gap: adding super-resolution to array tomography for correlated ultrastructural and molecular identification of electrical synapses at the *C. elegans* connectome. *Neurophotonics* 3:041802. 10.1117/1.NPh.3.4.041802 27175373 PMC4855082

[B56] Mastronarde D. N. (2005). Automated electron microscope tomography using robust prediction of specimen movements. *J. Struct. Biol.* 152 36–51. 10.1016/j.jsb.2005.07.007 16182563

[B57] McDonald K. (2007). *Cryopreparation Methods for Electron Microscopy of Selected Model Systems, Methods in Cell Biology.* Cambridge, MA: Academic Press, 23–56. 10.1016/S0091-679X(06)79002-1 17327151

[B58] McDonald K. Schwarz H. Muller-Reichert T. Webb R. Buser C. Morphew M. (2010). “Tips and tricks” for high-pressure freezing of model systems. *Methods Cell Biol.* 96 671–693. 10.1016/S0091-679X(10)96028-7 20869543

[B59] McDonald K. L. (2014). Out with the old and in with the new: rapid specimen preparation procedures for electron microscopy of sectioned biological material. *Protoplasma* 251 429–448. 10.1007/s00709-013-0575-y 24258967

[B60] McDonald K. L. Webb R. I. (2011). Freeze substitution in 3 hours or less. *J. Microsc.* 243 227–233. 10.1111/j.1365-2818.2011.03526.x 21827481

[B61] Meirovitch Y. Matveev A. Saribekyan H. Budden D. Rolnick D. Odor G. (2016). A multi-pass approach to large-scale connectomics. *arXiv:* 1612.02120 [Preprint].

[B62] Meng L. Mulcahy B. Cook S. J. Neubauer M. Wan A. Jin Y. (2015). The cell death pathway regulates synapse elimination through cleavage of gelsolin in *Caenorhabditis elegans* neurons. *Cell Rep.* 11 1737–1748. 10.1016/j.celrep.2015.05.031 26074078 PMC4481169

[B63] Mersey B. McCully M. E. (1978). Monitoring of the course of fixation of plant cells. *J. Microsc.* 114 49–76. 10.1111/j.1365-2818.1978.tb00116.x

[B64] Micheva K. D. Smith S. J. (2007). Array tomography: a new tool for imaging the molecular architecture and ultrastructure of neural circuits. *Neuron* 55 25–36. 10.1016/j.neuron.2007.06.014 17610815 PMC2080672

[B65] Mironov A. A. Polishchuk R. S. Beznoussenko G. V. (2008). *Chapter 5: Combined Video Fluorescence and 3D Electron Microscopy, Methods in Cell Biology.* Cambridge, MA: Academic Press, 83–95. 10.1016/S0091-679X(08)00405-6 18617029

[B66] Molina-Garcia L. Cook S. J. Kim B. Bonnington R. Sammut M. O’Shea J. (2018). A direct glia-to-neuron natural transdifferentiation ensures nimble sensory-motor coordination of male mating behaviour. *bioRxiv* 10.1101/285320

[B67] Moor H. (1987). “Theory and practice of high pressure freezing,” in *Cryotechniques in Biological Electron Microscopy*, eds Steinbrecht R. A. Zierold K. (Berlin: Springer), 175–191. 10.1007/978-3-642-72815-0_8

[B68] Moor H. Kistler J. Müller M. (1976). Freezing in a propane jet. *Experientia* 32:805.

[B69] Nguyen J. P. Shipley F. B. Linder A. N. Plummer G. S. Liu M. Setru S. U. (2016). Whole-brain calcium imaging with cellular resolution in freely behaving *Caenorhabditis elegans*. *Proc. Natl. Acad. Sci. U.S.A.* 113 E1074–E1081. 10.1073/pnas.1507110112 26712014 PMC4776509

[B70] Pereira L. Kratsios P. Serrano-Saiz E. Sheftel H. Mayo A. E. Hall D. H. (2015). A cellular and regulatory map of the cholinergic nervous system of *C. elegans*. *eLife* 4:e12432. 10.7554/eLife.12432 26705699 PMC4769160

[B71] Prevedel R. Yoon Y.-G. Hoffmann M. Pak N. Wetzstein G. Kato S. (2014). Simultaneous whole-animal 3D imaging of neuronal activity using light-field microscopy. *Nat. Methods* 11 727–730. 10.1038/nmeth.2964 24836920 PMC4100252

[B72] Randel N. Asadulina A. Bezares-Calderon L. A. Veraszto C. Williams E. A. Conzelmann M. (2014). Neuronal connectome of a sensory-motor circuit for visual navigation. *eLife* 3:e02730. 10.7554/eLife.02730 24867217 PMC4059887

[B73] Randel N. Shahidi R. Veraszto C. Bezares-Calderon L. A. Schmidt S. Jekely G. (2015). Inter-individual stereotypy of the *Platynereis* larval visual connectome. *eLife* 4:e08069. 10.7554/eLife.08069 26061864 PMC4477197

[B74] Riehle U. (1968). *Ueber die Vitrifizierung Verdünnter Wässriger Lösungen.* Doctoral dissertation, Zurich, ETH.

[B75] Rowley J. C. Moran D. T. (1975). A simple procedure for mounting wrinkle-free sections on formvar-coated slot grids. *Ultramicroscopy* 1 151–155. 10.1016/S0304-3991(75)80018-0 800684

[B76] Ryan K. Lu Z. Meinertzhagen I. A. (2016). The CNS connectome of a tadpole larva of *Ciona intestinalis* (L.) highlights sidedness in the brain of a chordate sibling. *eLife* 5:e16962. 10.7554/eLife.16962 27921996 PMC5140270

[B77] Ryan K. Lu Z. Meinertzhagen I. A. (2017). Circuit homology between decussating pathways in the *Ciona* larval CNS and the vertebrate startle-response pathway. *Curr. Biol.* 27 721–728. 10.1016/j.cub.2017.01.026 28216318

[B78] Saalfeld S. Cardona A. Hartenstein V. Tomancak P. (2009). CATMAID: collaborative annotation toolkit for massive amounts of image data. *Bioinformatics* 25 1984–1986. 10.1093/bioinformatics/btp266 19376822 PMC2712332

[B79] Saalfeld S. Cardona A. Hartenstein V. Tomancak P. (2010). As-rigid-as-possible mosaicking and serial section registration of large ssTEM datasets. *Bioinformatics* 26 i57–i63. 10.1093/bioinformatics/btq219 20529937 PMC2881403

[B80] Sammut M. Cook S. J. Nguyen K. C. Q. Felton T. Hall D. H. Emmons S. W. (2015). Glia-derived neurons are required for sex-specific learning in *C. elegans*. *Nature* 526 385–390. 10.1038/nature15700 26469050 PMC4650210

[B81] Sasakura H. Mori I. (2013). Behavioral plasticity, learning, and memory in *C. elegans*. *Curr. Opin. Neurobiol.* 23 92–99. 10.1016/j.conb.2012.09.005 23063296

[B82] Schalek R. Wilson A. Lichtman J. Josh M. Kasthuri N. Berger D. (2012). ATUM-based SEM for high-speed large-volume biological reconstructions. *Microsc. Microanal.* 18 572–573. 10.1017/S1431927612004710

[B83] Schrödel T. Prevedel R. Aumayr K. Zimmer M. Vaziri A. (2013). Brain-wide 3D imaging of neuronal activity in *Caenorhabditis elegans* with sculpted light. *Nat. Methods* 10 1013–1020. 10.1038/nmeth.2637 24013820

[B84] Seligman A. M. Wasserkrug H. L. Hanker J. S. (1966). A new staining method (OTO) for enhancing contrast of lipid-containing membranes and droplets in osmium tetroxide-fixed tissue with osmiophilic thiocarbohydrazide (TCH). *J. Cell Biol.* 30 424–432. 10.1083/jcb.30.2.424 4165523 PMC2106998

[B85] Serrano-Saiz E. Poole R. J. Felton T. Zhang F. De La Cruz E. D. Hobert O. (2013). Modular control of glutamatergic neuronal identity in *C. elegans* by distinct homeodomain proteins. *Cell* 155 659–673. 10.1016/j.cell.2013.09.052 24243022 PMC3855022

[B86] Simionescu N. Simionescu M. (1976). Galloylglucoses of low molecular weight as mordant in electron microscopy. I. Procedure, and evidence for mordanting effect. *J. Cell Biol.* 70 608–621. 10.1083/jcb.70.3.608 783172 PMC2109842

[B87] Simpson W. L. (1941). An experimental analysis of the Altmann technic of freezing-drying. *Anat. Rec.* 80 173–189. 10.1002/ar.1090800204

[B88] Smith J. E. Reese T. S. (1980). Use of aldehyde fixatives to determine the rate of synaptic transmitter release. *J. Exp. Biol.* 89 19–29. 6110693 10.1242/jeb.89.1.19

[B89] Steinbrecht R. A. (1985). Recrystallization and ice-crystal growth in a biological specimen, as shown by a simple freeze substitution method. *J. Microsc.* 140 41–46. 10.1111/j.1365-2818.1985.tb02658.x

[B90] Stevens J. K. Davis T. L. Friedman N. Sterling P. (1980). A systematic approach to reconstructing microcircuitry by electron microscopy of serial sections. *Brain Res. Rev.* 2 265–293. 10.1016/0165-0173(80)90010-7 6258704

[B91] Sulston J. E. Albertson D. G. Thomson J. N. (1980). The *Caenorhabditis elegans* male: postembryonic development of nongonadal structures. *Dev. Biol.* 78 542–576. 10.1016/0012-1606(80)90352-8 7409314

[B92] Sulston J. E. Horvitz H. R. (1977). Post-embryonic cell lineages of the nematode, *Caenorhabditis elegans*. *Dev. Biol.* 56 110–156. 10.1016/0012-1606(77)90158-0 838129

[B93] Sulston J. E. Schierenberg E. White J. G. Thomson J. N. (1983). The embryonic cell lineage of the nematode *Caenorhabditis elegans*. *Dev. Biol.* 100 64–119. 10.1016/0012-1606(83)90201-4 6684600

[B94] Szigeti B. Gleeson P. Vella M. Khayrulin S. Palyanov A. Hokanson J. (2014). OpenWorm: an open-science approach to modeling *Caenorhabditis elegans*. *Front. Comput. Neurosci.* 8:137. 10.3389/fncom.2014.00137 25404913 PMC4217485

[B95] Takemura S.-Y. Bharioke A. Lu Z. Nern A. Vitaladevuni S. Rivlin P. K. (2013). A visual motion detection circuit suggested by *Drosophila* connectomics. *Nature* 500 175–181. 10.1038/nature12450 23925240 PMC3799980

[B96] Towlson E. K. Vértes P. E. Ahnert S. E. Schafer W. R. Bullmore E. T. (2013). The rich club of the *C. elegans* neuronal connectome. *J. Neurosci.* 33 6380–6387. 10.1523/JNEUROSCI.3784-12.2013 23575836 PMC4104292

[B97] van Harreveld A. Crowell J. (1964). Electron microscopy after rapid freezing on a metal surface and substitution fixation. *Anat. Rec.* 149 381–385. 10.1002/ar.1091490307 14208983

[B98] Varshney L. R. Chen B. L. Paniagua E. Hall D. H. Chklovskii D. B. (2011). Structural properties of the *Caenorhabditis elegans* neuronal network. *PLoS Comput. Biol.* 7:e1001066. 10.1371/journal.pcbi.1001066 21304930 PMC3033362

[B99] Venkatachalam V. Ji N. Wang X. Clark C. Mitchell J. K. Klein M. (2016). Pan-neuronal imaging in roaming *Caenorhabditis elegans*. *Proc. Natl. Acad. Sci. U.S.A.* 113 E1082–E1088. 10.1073/pnas.1507109113 26711989 PMC4776525

[B100] Veraszto C. Ueda N. Bezares-Calderon L. A. Panzera A. Williams E. A. Shahidi R. (2017). Ciliomotor circuitry underlying whole-body coordination of ciliary activity in the *Platynereis* larva. *eLife* 6:e26000. 10.7554/eLife.26000 28508746 PMC5531833

[B101] Wagner R. C. (1976). The effect of tannic acid on electron images of capillary endothelial cell membranes. *J. Ultrastruct. Res.* 57 132–139. 10.1016/S0022-5320(76)80103-7 62845

[B102] Walther P. Ziegler A. (2002). Freeze substitution of high-pressure frozen samples: the visibility of biological membranes is improved when the substitution medium contains water. *J. Microsc.* 208 3–10. 10.1046/j.1365-2818.2002.01064.x 12366592

[B103] Ward S. Thomson N. White J. G. Brenner S. (1975). Electron microscopical reconstruction of the anterior sensory anatomy of the nematode *Caenorhabditis elegans*. *J. Comp. Neurol.* 160 313–337. 10.1002/cne.901600305 1112927

[B104] Ware R. W. Clark D. Crossland K. Russell R. L. (1975). The nerve ring of the nematode *Caenorhabditis elegans*: sensory input and motor output. *J. Comp. Neurol.* 162 71–110. 10.1002/cne.901620106

[B105] Webb R. I. Schieber N. L. (2018). “Volume scanning electron microscopy: serial block-face scanning electron microscopy focussed ion beam scanning electron microscopy,” in *Cellular Imaging: Electron Tomography and Related Techniques*, ed. Hanssen E. (Cham: Springer International Publishing), 117–148.

[B106] Weimer R. M. (2006). “Preservation of *C. elegans* tissue via high-pressure freezing and freeze-substitution for ultrastructural analysis and immunocytochemistry,” in *C. elegans: Methods and Applications*, ed. Strange K. (Totowa, NJ: Humana Press), 203–221. 10.1385/1-59745-151-7:203 16988436

[B107] Wells B. (1974). A convenient technique for the collection of ultra-thin serial sections. *Micron (*5 79–81. 10.1016/0047-7206(74)90035-1

[B108] Westfall J. A. Healy D. L. (1962). A water control device for mounting serial ultrathin sections. *Stain Technol.* 37 118–121. 10.3109/10520296209114587 14006339

[B109] White J. G. (2013). Getting into the mind of a worm–a personal view. *WormBook* 25 1–10. 10.1895/wormbook.1.158.1 23801597 PMC4781474

[B110] White J. G. Albertson D. G. Anness M. A. (1978). Connectivity changes in a class of motoneurone during the development of a nematode. *Nature* 271 764–766. 10.1038/271764a0 625347

[B111] White J. G. Southgate E. Thomson J. N. Brenner S. (1976). The structure of the ventral nerve cord of *Caenorhabditis elegans*. *Philos. Trans. R. Soc. Lond. B Biol. Sci.* 275 327–348. 10.1098/rstb.1976.0086 8806

[B112] White J. G. Southgate E. Thomson J. N. Brenner S. (1986). The structure of the nervous system of the nematode *Caenorhabditis elegans*. *Philos. Trans. R. Soc. Lond. B Biol. Sci.* 314 1–340. 10.1098/rstb.1986.0056 22462104

[B113] Williams E. A. Verasztó C. Jasek S. Conzelmann M. Shahidi R. Bauknecht P. (2017). Synaptic and peptidergic connectome of a neurosecretory center in the annelid brain. *eLife* 6:e26349. 10.7554/eLife.26349 29199953 PMC5747525

[B114] Yeh E. Kawano T. Ng S. Fetter R. Hung W. Wang Y. (2009). *Caenorhabditis elegans* innexins regulate active zone differentiation. *J. Neurosci.* 29 5207–5217. 10.1523/JNEUROSCI.0637-09.2009 19386917 PMC6665469

[B115] Zhang Y. Lu H. Bargmann C. I. (2005). Pathogenic bacteria induce aversive olfactory learning in *Caenorhabditis elegans*. *Nature* 438 179–184. 10.1038/nature04216 16281027

[B116] Zhen M. Samuel A. D. T. (2015). *C. elegans* locomotion: small circuits, complex functions. *Curr. Opin. Neurobiol.* 33 117–126. 10.1016/j.conb.2015.03.009 25845627

