# Corrigendum: A Pipeline for Volume Electron Microscopy of the *Caenorhabditis elegans* Nervous System

**DOI:** 10.3389/fncir.2019.00016

**Published:** 2019-03-20

**Authors:** Ben Mulcahy, Daniel Witvliet, Douglas Holmyard, James Mitchell, Andrew D. Chisholm, Yaron Meirovitch, Aravinthan D. T. Samuel, Mei Zhen

**Affiliations:** ^1^Lunenfeld-Tanenbaum Research Institute, Mount Sinai Hospital, Toronto, ON, Canada; ^2^Department of Molecular Genetics, University of Toronto, Toronto, ON, Canada; ^3^Department of Pathology and Laboratory Medicine, Mount Sinai Hospital, Toronto, ON, Canada; ^4^Nanoscale Biomedical Imaging Facility, The Hospital for Sick Children, Peter Gilgan Centre for Research and Learning, Toronto, ON, Canada; ^5^Center for Brain Science, Department of Physics, Harvard University, Cambridge, MA, United States; ^6^Section of Cell and Developmental Biology, Division of Biological Sciences, University of California, San Diego, La Jolla, CA, United States; ^7^Computer Science and Artificial Intelligence Laboratory, MIT, Cambridge, MA, United States; ^8^Department of Physiology, University of Toronto, Toronto, ON, Canada; ^9^Department of Cell and Systems Biology, University of Toronto, Toronto, ON, Canada

**Keywords:** *C. elegans*, volume electron microscopy, connectome, nervous system, high-pressure freezing

“Yaron Meirovitch” was not included as an author in the published article.

The authors apologize for this error and state that this does not change the scientific conclusions of the article in any way. The original article has been updated.

